# Impact of ground surface subsidence caused by underground coal mining on natural gas pipeline

**DOI:** 10.1038/s41598-023-46814-5

**Published:** 2023-11-07

**Authors:** Oleg Bazaluk, Oleksandr Kuchyn, Pavlo Saik, Saule Soltabayeva, Hanna Brui, Vasyl Lozynskyi, Oleksii Cherniaiev

**Affiliations:** 1 Belt and Road Initiative Center for Chinese-European Studies (BRICCES), Guangdong University of Petrochemical Technology, Maoming, 525000 China; 2 Department of Geodesy, Dnipro University of Technology, Dnipro, 49005 Ukraine; 3 Department of Mining Engineering and Education, Dnipro University of Technology, Dnipro, 49005 Ukraine; 4 Department of Surveying and Geodesy, Satbayev University, 50013 Almaty, Kazakhstan; 5 Department of Surface Mining, Dnipro University of Technology, Dnipro, 49005 Ukraine

**Keywords:** Environmental sciences, Engineering

## Abstract

Underground mining of minerals is accompanied by a change in the rock mass geomechanical situation. This leads to the redistribution of stresses in it and the occurrence of unexpected displacements and deformations of the earth's surface. A significant part of the civil and industrial infrastructure facilities are located within the mine sites, where mining and tunneling operations are constantly conducted. Irrational planning of mining operations can lead to loss of stability and destruction of undermined facilities. Therefore, it is important to study the earth’s surface deformation processes during mining operations, which ensures safe and sustainable operating conditions. The research objective of this paper is to analyse the behaviour of a natural gas pipeline under the influence of underground mining activities, with a particular focus on understanding the effects of horizontal surface deformations and their potential impact on pipeline safety and structural integrity. Its performance and safety are determined on the basis of the found parameters of the earth's surface horizontal deformations and their comparison with permissible parameters characterizing the conditions for laying pipelines, depending on the mining-geological conditions and the degree of their undermining. Based on determined conditions for the safe undermining of the natural gas pipeline, it has been revealed that in its section between the PK212+40 and PK213+80 (140 m) pickets, the estimated parameters of the earth's surface horizontal deformations exceed their permissible values. This can cause deformation and damage to the pipeline. For the safe operation of the pipeline during the period of its undermining, in order to eliminate the hazardous impact of mining the longwall face, additional protection measures must be applied. It is therefore recommended that the gas pipeline between the PK212 and PK214+20 pickets be opened prior to the displacement process (200 m from the stoping face), thus reducing the density of the gas pipeline-soil system. Recommendations for controlling the earth’s surface deformations within the natural gas pipeline route are also proposed, which will ensure premature detection of the negative impact of mining operations.

## Introduction

Technogenic activity in the mining of minerals leads to a redistribution of the rock mass stresses and the formation of displacements and deformations of the earth's surface, which have a harmful effect on civil and industrial infrastructure facilities^1–3^. In Ukraine, the normative document regulating the conditions for the safe undermining of objects located on the earth's surface is the “Regulations of undermining of buildings, structures and natural objects during underground coal mining”^4^. In accordance with this document, the boundaries of the mining impact zones on the earth's surface objects, the estimated and permissible deformations for undermined objects are determined, and requirements are set for the rational extraction of coal and the application of measures to protect undermined objects from the impact of mining operations. An increase in the depth of mining^5–7^, the reactivation of mineral reserves from protecting pillars under densely built-up areas^8,9^ leads to an expansion of the territories involved in the process of the earth's surface displacement.

**Figure 1:**
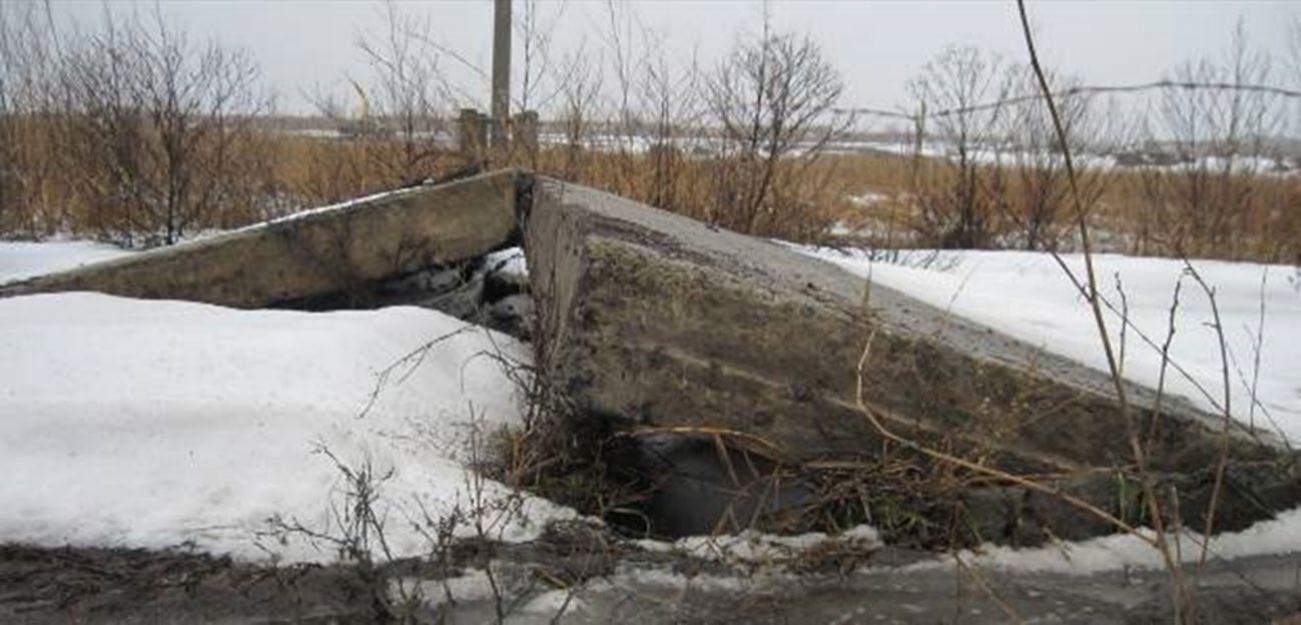
View of the damaged road surface due to the impact of mining operations (automobile road above the underground mine).

Coal mining plays a decisive role in the development of the power industry and the state economy of Ukraine^10–12^ and other developed countries^13–16^. In this case underground coal mining without the use of backfilling technologies has led to serious changes in the earth's surface^17^. Figure 1 shows a visual fixation of the influence of the earth's surface deformations during mining operations on the reinforced-concrete pavement of the road passing within the mine field of PSD Dniprovska mine, PJSC DTEK Pavlohradvuhillya (Ukraine). As a result of this influence, the concrete blocks rose by 50 cm from their previous position.

All industrial, agricultural, transport, energy, hydro-technical and other structures, residential and public buildings, water bodies, watercourses, healing springs and mud, natural, historical and cultural monuments, lands, forests, green spaces and other objects are subject to mandatory protection from harmful impact of mining operations^18–20^. If this impact can cause obstacles to the normal operation or use of the above-mentioned objects for their intended purpose, and demolition or transfer outside the impact zone of mine workings is impossible or it is not economically feasible, mining is not performed.

Surface subsidence is a common issue that can occur during underground coal mining^21–24^. There are several measures that can be taken to eliminate or reduce surface subsidence. The process of backfilling and grouting can help to eliminate or reduce surface subsidence by filling the cavities created by underground mining activities^25,26^. This involves injecting backfilling materials or other materials into the goaf to stabilize the ground. Various subsidence control methods such as hydraulic stowing, hydraulic fracturing, and longwall caving can be used to minimize subsidence^27^. A proper mine design can help to reduce the impact of underground mining activities on the surface^28,29^. This can involve using pillars or leaving unmined areas to provide support for the overlying rock. Surface and groundwater management can help to reduce surface subsidence by controlling the amount of water that enters the subsurface^23,30^. This can be achieved through the use of dewatering techniques and drainage systems. Monitoring and analyzing the subsurface conditions can help to detect subsidence and take corrective action^31,32^. This can involve monitoring the groundwater levels and subsurface movements using various techniques such as GPS and remote sensing.

Overall, a combination of these measures can help to reduce surface subsidence during underground coal mining activities but not eliminate it.

The research object of this paper is a natural gas pipeline laid within the mine field boundaries. Damage to the natural gas pipeline elements can cause fires, explosions, equipment failure, injury and death of people. External gas pipelines on the territory of settlements are laid underground in accordance with the requirements of DBN B.2.2.-12. Aboveground or surface-mounted laying of external steel pipelines is allowed inside residential areas, on the route sections along the streets if underground laying is impossible due to density of underground communications, the presence of rocky soils that come to the surface, as well as when crossing natural obstacles (rivers, ravines, gully, etc.) by gas pipelines. The route and pipe material (steel or polyethylene) of underground gas pipelines are selected based on the corrosive aggressiveness of soils, the presence of stray currents, etc.

The choice of measures to protect the gas transportation system, which is within the possible mining impact boundaries, provides for:

- Determining the boundaries of the impact zone of mine workings and the duration of the earth's surface displacement process;
- Determining of estimated and permissible earth's surface deformation parameters for objects located in the impact zone of mining operations;
- Setting the requirements for the rational mining of minerals in terms of the application of measures to protect undermined objects from the impact of mining operations.

The pipelines of the gas transportation system are prone to deformations, although they are laid at a considerable distance from mining operations. The earth's surface horizontal deformations have the most dangerous impact on the performance of gas pipelines and lead to an increase in the risk of accidents. The following are some of the possible harmful effects of the earth's surface displacement process on natural gas pipelines:

- Change in the planned position of the topographic (earth) surface: as a result of mining operations along the gas pipeline route, vertical and horizontal displacements, as well as deformations of the earth's surface occur. This can lead to nonuniform horizontal loads on the gas pipeline metal structure, which in turn can lead to gas pipeline damage and the occurrence of gas leaks. As noted in the work of^33^, conducting of mining operations leads to the development of additional loads and displacements of gas pipelines. In the case of gas pipelines, the influence of deformations in the longitudinal direction is of primary importance. Longitudinal forces caused by friction when the rock mass moves along the external surface of the pipeline, taps, tees, fittings, flanges and expansion joints can lead to damage to the gas pipeline. Depending on the type of soil and its density, the harmful effect of deformations can change towards deterioration or vice versa.
- The possibility of bench formation: as a result of mining operations, the rock mass is exposed to alternating dynamic loads, which can lead to the formation of benches on the earth’s surface.
- Gas pipeline geometry disturbance: due to the earth's surface deformation, the gas pipeline geometry may be disturbed. This can lead to nonuniform pressure in some gas pipeline sections and its subsequent damage.
- The influence of tensile and compression deformations: tensile deformation has a harmful effect on welded seams and, with a high density of the soil laid, can lead to a pipe rupture. Compression deformations are conventionally less harmful, but may cause geometric deformation of the gas pipeline in the vertical or horizontal plane.

**Figure 2:**
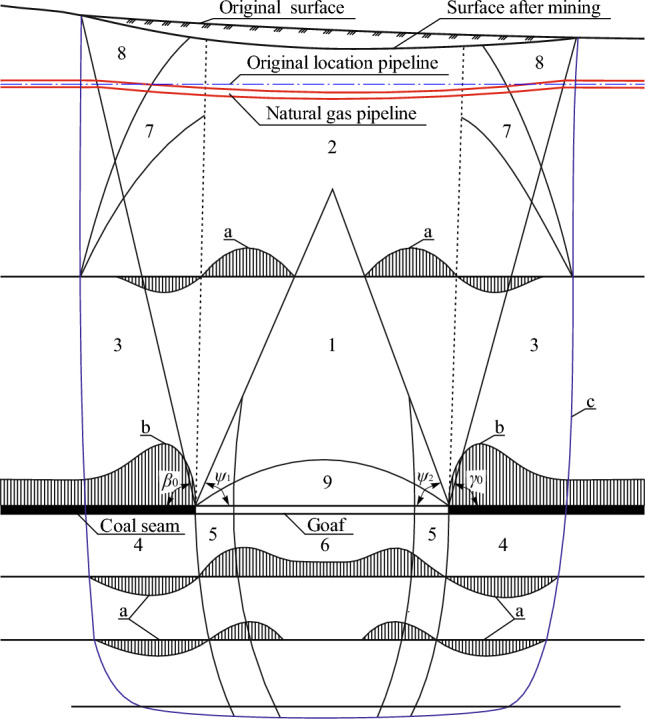
Formation of a shift trough during underground mining of coal reserves: (**a**)—graphs of the rock mass vertical deformations; (**b**)—normal stress curves; (**c**)—boundary of the rocks mass displacement zone; 1—zone of complete displacements; 2—zone of greatest bending of layers; 3,4—compression zones; 5—seam bottom heaving zone; 6—de-stressing zone; 7—zone of small vertical tensile and compression deformations; 8—displacement zone above the seam edge, 9—zone of caving; *Ψ*_1_ and *Ψ*_2_—angles of complete displacements, deg; *β*_0_—boundary angle to the dip; *γ*_0_—boundary to the rise.

In a general view, the processes of changing stress–strain state of rocks under the impact of mining the stratified deposits are shown in Fig. 2.

Under the influence of stope operations, the earth's surface and the objects located on it (in our case, a gas pipeline) fall into the shift trough. This is the earth's surface area on which, under the impact of underground mining method, horizontal and vertical displacements and deformations (settlement, slope, curvature, horizontal displacements and deformations) occur. Its boundary on the earth's surface is determined by the boundary angles.

The stability of deepened pipelines influenced by surface deformations, landslides, faults during tunnel construction and underground mining of deposits is studied in sufficient detail in the works^34–41^. Based on numerical modeling, laboratory tests and field studies, these studies have determined the parameters of pipeline interaction with a rock mass. For example, in the work^42^, it is recommended when designing a pipeline or selecting the trajectory of laying it in the mining zone, to choose a direction parallel to the strike or inclined direction of mining in order to avoid dangerous (complicated) deformations. To predict the deformations and stresses of a deepened pipeline in the subsidence zone caused by mining operations, the authors^43^ propose to use an analytical method based on the assumption that the pipeline bending curve is described by the rock mass deformation, as provided by the probability function integration method. The stress distribution in the laid pipeline, calculated by the proposed method, is consistent with the finite element model, and this method is suitable for studying deformation and stresses for pipelines laid across the direction of conducted stope. In the work^44^, it has been revealed that the main factor in the occurrence of stresses in the pipeline is horizontal deformation and vertical curvature. This led to the development of a methodology for determining the values and places of maximum tensile and compression stresses on the pipeline and recommendations for partial or complete protection to prevent possible damage to the pipeline.

The conducted research provides important background information for assessing the state of pipelines during underground mining operations, but it should be noted that the results of using analytical methods and mathematical modeling methods do not always coincide with the results of monitoring the actual values of the earth's surface displacements and deformations. The most reliable, from the point of view of the accuracy of determining their predictive values, are the results of instrumental observations, which can be used as boundary when modeling the displacement process.

The purpose of the paper is to present the influence of rock mass deformations occurring in mining areas on natural gas pipelines. Based on the purpose set in the work, it is necessary to determine the boundaries of the mining impact on the earth's surface and the length of semi-troughs. After that, it is necessary to study the parameters of the earth's surface expected displacements and deformations, as well as their influence on the gas pipeline laid within the mine field; propose recommendations for managing the earth's surface deformations during mining operations. An important component of our research is the prediction of surface deformation parameters, which is essential for better understanding the impact of underground mining activities on the gas pipeline safety and structural integrity.

## Materials and methods

### Research object characteristics

The natural gas pipeline is made welded from pipes with a diameter of 325 mm and a wall thickness of 6 mm made of steel St. 20 (temporary tensile strength of steel is 420 MPa, yield strength of steel is 245 MPa). The length of the gas pipeline within the studied area is 709 m. The design maximum pressure in the gas pipeline branch is 5.5 MPa. The depth of laying the gas pipeline is 1.0 m up the pipe, and the backfill soil is a hard loess-like loam. The gas pipeline is protected against soil corrosion by an imported film with a thickness of 0.63 mm and cathodic protection. The gas pipeline is designed with 100% control of all welded joints.

The main gas pipeline section, in accordance with the mining development program, will be undermined with the longwall face No. 1065, which mines the coal seam c_10_^v^ within the boundaries of the PSD Dniprovska mine, PJSC DTEK Pavlohradvuhillya (Ukraine). The stoping face mining-geological characteristics are given in Table 1.

**Table 1:** Mining and geological characteristics of stoping faces.

Seq no	Parameter	Value (longwall face No. 1065)
1	Average mining depth, (*H*), m	394
2	Sediment thickness (covering deposits), (*h*), m	84
3	Panel length, (*D*_1_), m	1127
4	Stoping faces (longwall face) length, (*D*_2_), m	254
5	Coal seam dip angle (*α*), deg	4
6	Seam c_10_^v^ thickness (*m*), m	1.27

The coal reserves are mined with a long panel to the coal seam rise. The roof management method is complete caving.

### Determining the boundaries of the mining impact zone and the lengths of semi- troughs

It is possible to determine the boundaries of the impact zone of the stope operations graphically by the boundary angles in the section across the strike and along the strike. Boundary angles are external angles relative to the goaf, formed on vertical sections of the trough main sections by horizontal lines (sequentially drawn in bedrock and sediments) connecting the mine working boundary with the boundary of its impact zone on the earth's surface. For the Western Donbass conditions, the values of the boundary angles of the bedrock and cover rock are:

- Along the strike is *δ*_0_—65°;
- To the dip is *β*_0_—65°;
- To the rise – *γ*_0_—65°;
- In sediment is *φ*_0_—45°;
- Maximum draw angle is* θ*—90°–0.8*α*.

The boundaries of semi-troughs are determined on the main intersections of the shift trough using the angles of total displacements, the values of which for the given conditions are equal to:

- To the dip of the seam is *ψ*_1_—55°;
- To the rise of the seam is *ψ*_2_—55° + 0.3*α* = 56.2°;
- To the dip of the seam is *ψ*_3_—55°.

**Figure 3:**
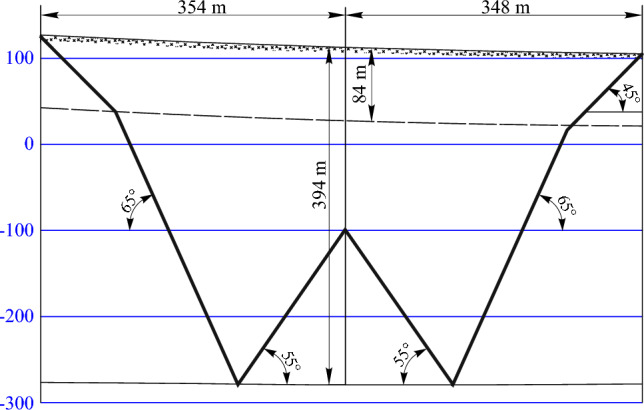
Determining the impact zone boundaries and the lengths of semi-troughs on the main intersection along the strike.

**Figure 4:**
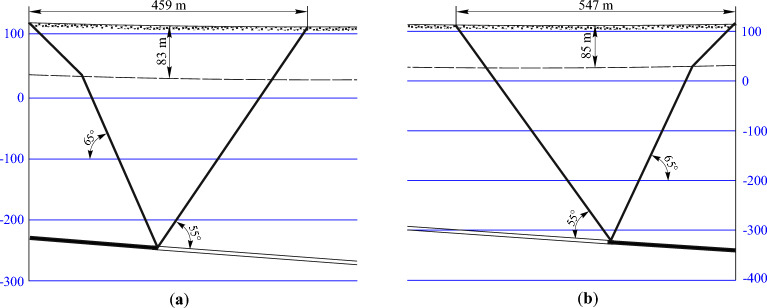
Determining the impact zone boundaries and the lengths of semi-troughs on the main intersection from the side of: (**a**) the seam dip; (**b**) the seam rise.

The construction of the impact zone boundaries and setting the lengths of the semi-troughs depending on the influence of the longwall face No. 1065 on the main intersection along the strike are shown in Fig. 3, on the main intersections across the strike—in Fig. 4.

According to the results of graphic constructions, the boundary of the impact zone of mining operations when mining the coal seam with longwall face No. 1065, which mines the coal seam c_10_^v^, is 702 m along its strike and 1585 m across the strike.

### Determining the main displacement process parameters (the earth's surface expected displacements and deformations)

For the Western Donbass conditions (mining of gas grade coal seams), the relative maximum subsidence value is calculated according to the following formula:

1 $$q_{0} = q_{0}^{{\prime }} \left[ {1 + \left( {1 - q_{0}^{{\prime }} } \right)\frac{{H_{1} }}{H}} \right],$$

where $q_{0}^{{\prime }}$ is a dimensionless value, determined by Ref.^4^ and is equal to 0.85; *H* is average depth of undermining; *H*_1_ is the distance from the earth's surface to a previously mined seam.

The maximum ground surface subsidence is determined by the formula:

2 $$\eta_{m} = q_{0} m\cos \alpha N_{1} N_{2} ,$$

where $q_{0}$ is value of relative maximum subsidence; *m* is value of uniform lowering of the mine working upper part contour; *α* is seam occurrence angle; *N*_1_, *N*_2_ is conditional coefficients characterizing the degree of the earth’s surface undermining, across the strike and along the strike, respectively, dimensionless values.

Coefficients *N*_1_, *N*_2_ for coal deposits are calculated using the following formulas:

3 $$N_{1} = \sqrt {0.9\left( {\frac{{D_{1} }}{H} + \Delta D_{dip} + \Delta D_{rise} } \right)} ,$$

4 $$N_{2} = \sqrt {0.9\left( {\frac{{D_{2} }}{H} + \Delta D_{str} + \Delta D_{acrs} } \right)} ,$$

where *D*_1_, *D*_2_ are the mine working length, across the strike and along the strike, respectively; ∆*D*_*dip*_ is correction to the relative length of the longwall face due to the pillar from the side of the dip; ∆*D*_*rise*_ is correction to the relative length of the longwall face due to the pillar from the side of the rise; ∆*D*_*str*_ is correction to the relative length of the longwall face due to the pillar from the side of the strike; ∆*D*_*acrs*_ is correction to the relative length of the longwall face due to the pillar from the side of across the strike.

The parameters of the earth’s surface displacement and deformation processes when mining the longwall face No. 1065 of the seam c_10_^v^ are given in Table 2.

**Table 2:** Displacement parameters when mining the longwall face No. 1065 of the seam c_10_^v^.

Parameter	Value
Dimension *D*_1_, m	1127
Dimension *D*_2_, m	254
Dip angle, deg	4
Average depth, m	394
Seam thickness, m	1.27
Sediment thickness, m	84
Coal grade	D-G
Boundary angle, deg:	
To the dip	65
To the rise	65
Along the strike	65
In sediments	45
Angles of complete displacements, deg:	
To the dip	55
To the rise	56
Along the strike	55
Relative maximum subsidence	0.8
Primary undermining	Done
Primary undermining depth, *H*_1_	0
Face movement direction	To the dip
Maximum subsidence	0.74
Coefficients of undermining:	
To the dip	547
To the rise	459
Along the strike	346
Corrections ΔD:	
To the dip	− 0.101
To the rise	0.02
Along the strike	− 0.101
Across the strike	0.05

The earth's surface expected displacements and deformations are set using the “Undermining” software product. It takes into account the requirements of the state standard of Ukraine^4^ and allows determining the parameters of horizontal displacements and deformations of the earth's surface along the strike and across the strike. This software was developed by the author of this paper, Doctor of Technical Sciences O.S. Kuchyn.

**Figure 5:**
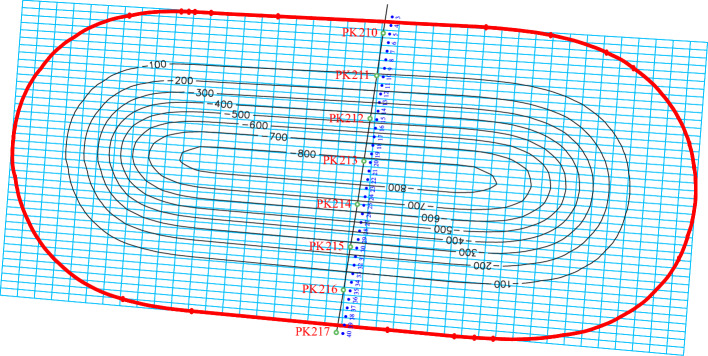
Formation of the calculation grid and isolines (mm) of the ground surface subsidence.

The mining-geological characteristics of the studied area and the stoping face parameters serve as the initial data for conducting the research. The introduction of the above data makes it possible to construct spatial models of the seam surface, sediments and the earth's surface, as well as to take into account the change in the hypsometry of these surfaces. The ground surface subsidences within the shift trough are calculated in grid vertices and conditional coordinates (Fig. 5).

The values of displacements and deformations of the earth's surface are also determined in the nodes of the calculation grid. The number of nodes in each direction (along the strike and across the strike) is 40. Isolines of the ground subsidence are constructed by interpolating the subsidence values at the calculation grid nodes.

### Determining the parameters of the earth's surface deformations, affecting the safety of the gas pipeline operation

The safe operation of gas pipelines in the area of mining operations is based on a comparison of permissible and determined earth's surface horizontal deformation parameters in the mining impact zone^4^.

The permissible parameter of the earth's surface horizontal deformations in the mining impact zone for gas pipelines is determined by the formula:

5 $$\left[ \varepsilon \right] = \left[ \varepsilon \right]_{n} \cdot k_{p} ,$$

where $\left[ \varepsilon \right]_{n}$ is normative permissible relative deformation parameter, which, according to the mining-geological conditions for laying the gas pipelines with a temporary tensile strength of steel of 420 MPa and a steel yield strength of 245 MPa, is taken as $\left[ \varepsilon \right]_{n}$ 2.5 × 10^−3^; *k*_*p*_ is the coefficient of residual deformation resource of gas pipelines. In this case, the permissible parameter of horizontal deformations, depending on the conditions of laying the gas pipeline and its characteristics (soil, yield strength, temporary tensile strength) vary within (1.5 × 10^−3^–3.5 × 10^−3^).

The coefficient of the residual deformation gas pipeline resource *k*_*p*_ is determined by the formula:

6 $$k_{p} = \frac{{\left[ \varepsilon \right]_{n} - \left| {\varepsilon_{0} } \right|}}{{\left[ \varepsilon \right]_{n} }},$$

where *ε*_0_ is the estimated parameter of horizontal deformations caused by the impact of past undermining, determined by the formula:

7 $$\varepsilon_{0} = \sum\limits_{1}^{N} {\varepsilon_{0n} \mu_{\Pi n} } ,$$

where ε_0n_ is estimated horizontal deformations from one n-th stope during past undermining; *n* is the mine working number in the case of past undermining in the order of its mining; *N* is quantity of mine workings in past undermining; *μ*_*Пn*_ is the coefficient of gas pipeline adaptation to deformation effects from past undermining, taken depending on the gap in time Tn between the end of the *n*-th past undermining and the beginning of the planned undermining.

In the absence of preliminary undermining of the earth's surface by mining operations, the estimated parameter of horizontal deformations is *ε*_0_ = 0. Therefore, for given mining-geological conditions of laying the gas pipeline $\left[ \varepsilon \right] = \left[ \varepsilon \right]_{n}$ = 2.5 × 10^−3^.

Given the results of numerical calculations of the earth's surface horizontal deformations (Subsection “Determining the main displacement process parameters (the earth's surface expected displacements and deformations”) and coefficients of operating conditions for gas pipelines and adaptation to deformation effects, the horizontal deformation parameter is determined using the formula:

8 $$\varepsilon = m_{\varepsilon } \left| {\varepsilon_{\max } } \right|\mu_{n} ,$$

where $m_{\varepsilon }$ is coefficient of operating conditions for gas pipelines (taken equal to 1); $\varepsilon_{\max }$ is horizontal deformations based on the results of numerical calculations; $\mu_{n}$ is coefficient of adaptation of gas pipelines to deformation effects, taken equal to 1.

## Results and discussion

**Figure 6:**
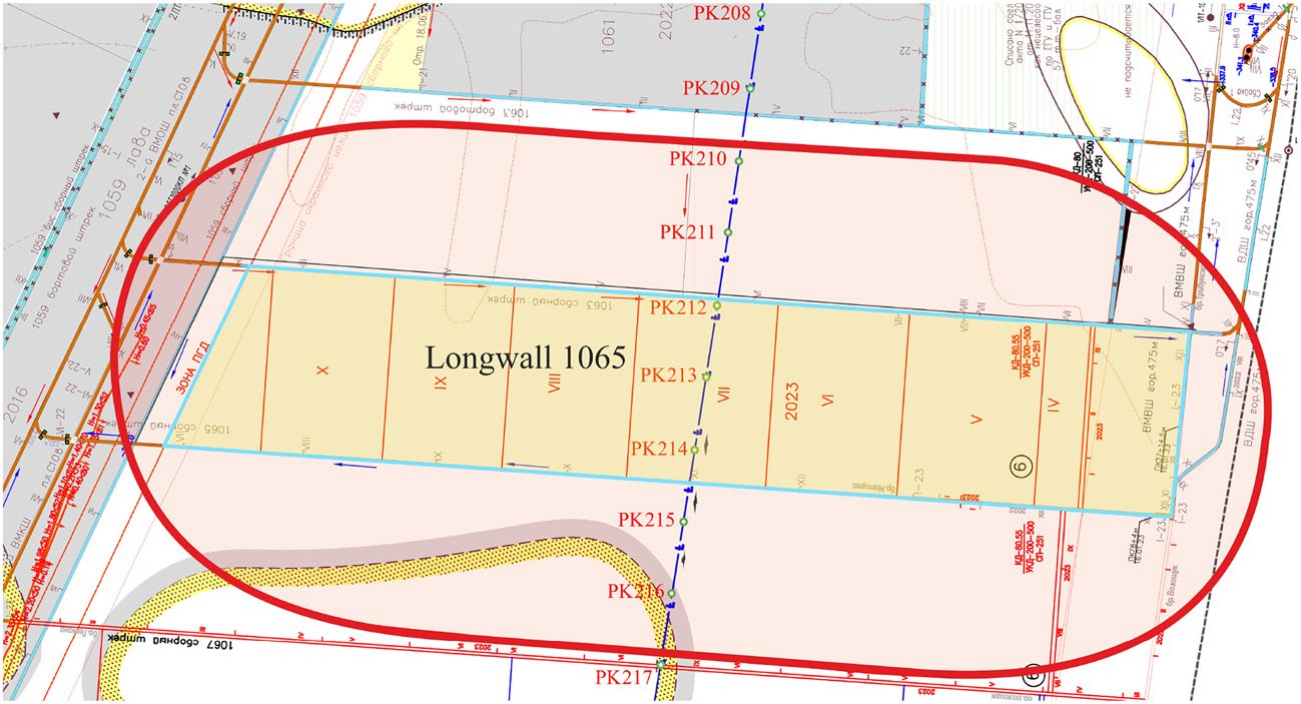
The impact zone boundary of the stope operations in the longwall face No. 1065 of the seam c_10_^v^, combined with the calendar plan for conducting mining operations.

In accordance with the mining-geological and mining-technical conditions for conducting stope operations, vertical sections are built in the main shift trough intersections, as well as the boundaries of the impact zone of stope operations in the longwall face No. 1065 of the seam c_10_^v^ are determined in accordance with the mining plan (Fig. 6).

**Figure 7:**
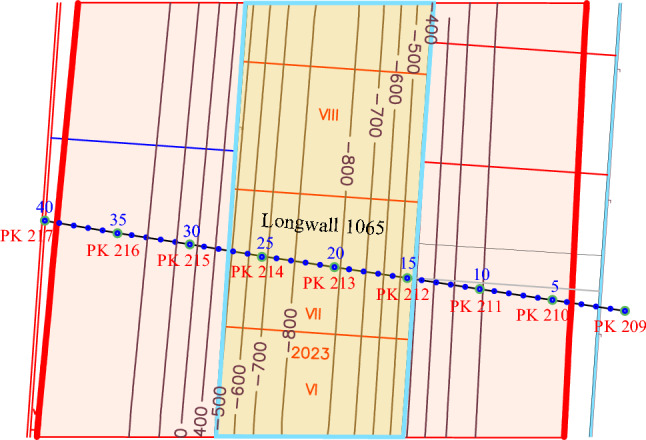
Positions of the test points and the gas pipeline pickets within the impact zone.

The total area of the undermined earth's surface is 99.8 hectares. A gas pipeline section falls into the impact zone of stope operations in the town of Petropavlivka, Dnipropetrovsk oblast, under hydraulic fracturing of seams in the city of Ternivka (209-217 pickets). The pipeline section (PK 209-217) is divided into test points with an interval of 20 m. About 30 m of the PK209-210 gas pipeline and 80 m of PK216-217 gas pipeline fall into the studied area. The total length of the undermined gas pipeline section is 710 m. The positions of the test points of the undermined gas pipeline section and the isolines (mm) of the ground surface expected subsidence are shown in Fig. 7.

Having received data on the degree of impact of stope operations within the specified boundaries, the earth's surface expected displacements and deformations along the undermined gas pipeline section have been determined in accordance with subsection “Determining the main displacement process parameters (the earth's surface expected displacements and deformations)” and based on the application of the coefficients of typical curves. The coefficients were selected based on long-term mine surveying instrumental observations, including those in the Western Donbass from 1963 to 2012. During this time, 35 observation stations have been laid, consisting of 76 profile lines and 3120 ground benchmarks. In total, more than 500 series of observations have been conducted^45,46^.

The ratio of the cover rock strength to the average mining depth within the longwall face No. 1065 is *h*/*H*_*av*_ = 83/394 = 0.21. According to^4^, the relative maximum ground surface subsidence during the primary undermining is equal to *q*_0_ – 0.8, and the relative horizontal displacement is *a*_0_ – 0.3. The coefficient of undermining the earth's surface across the strike is *N*_1_ – 1, along the strike is *N*_2_ – 0.732. According to formula (1), the maximum subsidence value is $\eta_{m}$ is 741 mm. At the same time, it has been revealed in the earlier conducted research^47^ that under the conditions of mining sloping weakly metamorphosed coal seams in the Western Donbass, the relative maximum ground surface subsidence value is underestimated. This value underestimation is also confirmed by the results of mine surveying instrumental observations^48^.

The value of the relative maximum ground surface subsidence (*q*_0_), according to earlier conducted research, given in the materials of the doctoral dissertation of Kuchyn O.S. in similar studied areas in the conditions of primary undermining was 0.9. Thus, according to formula (1), the maximum subsidence value for our conditions is 0.834 m. This value can be used as a boundary when determining the conditions for the safe undermining of the earth's surface and the gas pipeline.

**Figure 8:**
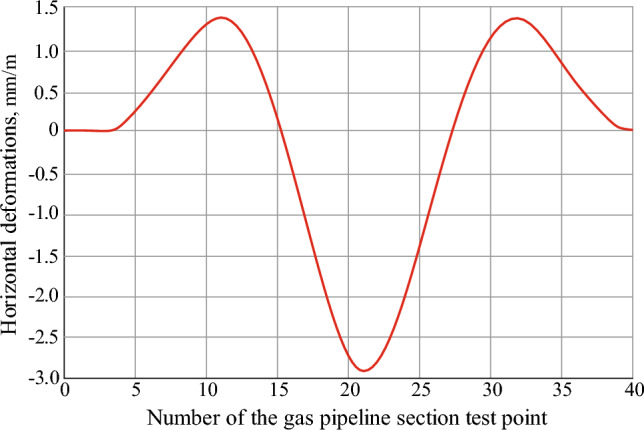
Graph of the earth's surface horizontal deformations in the test points along the gas pipeline route due to the influence of the longwall face No. 1065 of the seam c_10_^v^.

The determined maximum ground surface subsidence is 12% more than the theoretically calculated one, which is taken for research in the environment of the “Undermining” software product. Based on the conducted research results, the dependence of the change in the earth's surface horizontal deformations in the test points along the gas pipeline route on the influence of the longwall face No. 1065 of the seam c_10_^v^ has been obtained (Fig. 8).

The results of predicting the earth’s surface expected horizontal deformations show that the compression values (Fig. 8 with the sign “-”) are twice as high as the tension values. This is the result of incomplete undermining of the earth's surface along the strike line of the seam. The graph of horizontal deformations is symmetrical due to the location of the gas pipeline route almost parallel to the direction of the strike line of the seam.

**Figure 9:**
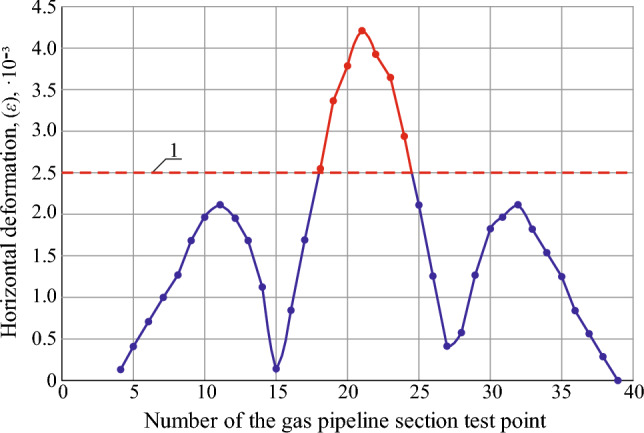
Graph of changes in the horizontal deformation parameters in the studied points along the gas pipeline route due to the influence of the longwall face No. 1065 of the seam c_10_: 1—boundary normative permissible relative deformation.

A condition for the safe gas pipeline operation is compliance with permissible earth's surface deformation parameters^5^. For the given mining-geological conditions, the value of this parameter is 2.5 × 10^−3^. In accordance with the found boundaries of the impact zone of stope operations, the maximum earth's surface deformation parameters are studied. The gas pipeline is laid parallel to the stoping face line. Under this condition, the earth's surface maximum deformations are observed only after the end of the displacement process. The graph of horizontal deformation parameters in the studied points along the gas pipeline route due to the influence of the longwall face No. 1065 of the seam c_10_^v^ is shown in Fig. 9.

The graph shown in Fig. 9 characterizes the impact of mining operations on the earth's surface horizontal deformation. Namely, the horizontal deformation parameter, exceeding the permissible values of 2.5 × 10^−3^, is located between the PK212+40 and PK213+80 (140 m) pickets. In the center of the studied area, there is a manifestation of the earth’s surface dangerous compressions, which can lead to the pipeline bending and a change in its operating conditions. Thus, based on the analysis of the data shown in Fig. 9, it is necessary to open the section of the gas pipeline that are located in the zone of horizontal deformations that exceed the permissible values. This will ensure the safe operation of the pipeline during the period of its exploitation between pickets PK212 and PK214+20 before the start of the displacement process (at a distance of 200 m from the stoping face.

Below are presented recommendations for controlling the earth's surface deformations when conducting mining operations.

High-pressure gas pipeline undermining, according to “Regulations of undermining”^4^, should be controlled by instrumental observations and provide for the possibility of applying operational protective measures. For this purpose, it is necessary to lay down observation stations within the impact zone of the longwall face No. 1065 of the seam c_10_^v^ along the gas pipeline route. The ground benchmarks of the observation station are located within the impact zone of the longwall face No. 1065 of the seam c_10_^v^, and the reference ones are located outside it at a distance of 50 and 100 m. In this case, the profile line total length is 1040 m.

**Figure 10:**
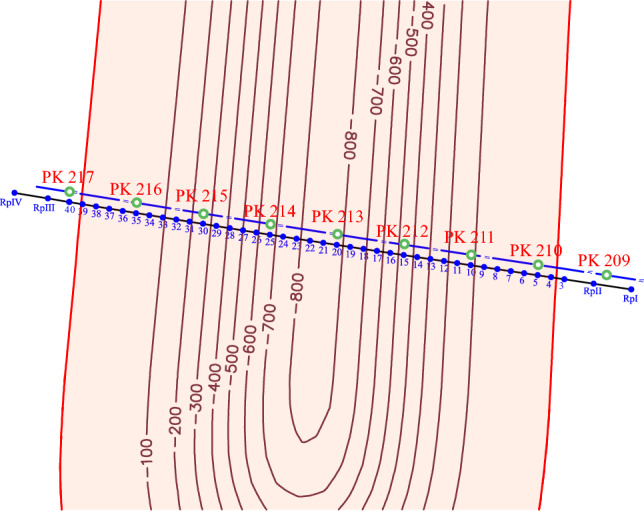
Plan for laying down ground and reference benchmarks within the studied area of the mine field.

The observation station benchmarks, depending on the possibility of their long-term preservation, may or may not be deepened. The reference benchmarks are concreted to the entire depth of their setting. Ground benchmarks are made from metal rods 1.5 m long and 20 mm in diameter. In the upper ends of the benchmarks, recesses 12 mm in diameter are drilled or thrown on. According to^42^, they are laid 20 m apart along lines located at a distance of 15 m from the projection of the test points (Fig. 10, points 3–40) of the gas pipeline axis on the earth's surface. The observation station reference benchmarks are laid outside the impact of mining operations (at the beginning and at the end of the profile line) with two ground benchmarks at distances of at least 50 m and 100 m from the beginning and end of the profile line, respectively.

The RpI and RpII reference benchmarks should be laid down from the side of mining out the longwall face No. 1065 at PK208+20 and PK209+20 pickets, respectively. On the other hand, the project provides for the laying down of the RpIII and RpIV reference benchmarks at (PK217+50 and PK218+50). Total number of the profile line working benchmarks is 42.

The positions of the observation station reference and working benchmarks are removed according to the classical method or using a GNSS-receiver in RTK mode^49,50^. Altitude referencing of the observation station reference benchmarks is performed according to the IV class leveling method from the initial benchmarks located on the industrial sites of the mining enterprise.

Laying and initial measurements at the observation station should be performed before the beginning of the impact of mining operations on the gas pipeline and, accordingly, on the working benchmarks. The displacement process along the gas pipeline trajectory can begin when the stoping face of the longwall face No. 1065 approaches at a distance of 184 m, which corresponds to approximately 1.2 months. Under such conditions, it is necessary to lay down benchmarks and perform the first observation 2 months before the start of the displacement process (the position of the stoping face at a distance of about 300 m from the gas pipeline route).

The first observation should be conducted no less than 3 days after the laying of the benchmarks (time for stabilization of the driven benchmarks). A control measurement should be made in a week. The time interval between instrumental observations during the displacement period is set depending on the monthly rate of the ground surface subsidence.

In our case, the maximum ground surface subsidence is 834 mm, the average depth of undermining is 394 m, and the face advance rate is 150 m/month or 5 m/day. For these conditions, the maximum estimated daily subsidence rate is 22 mm/day.

A natural gas pipeline is a potentially dangerous object, therefore, during the displacement process active stage (2.5 months), it is recommend to maintain the time interval between instrumental measurements—once a month. After the end of the displacement process active stage (4.3 months), the periodicity of observations can be increased up to 2 months.

## Conclusions

This paper presents the results of determining the conditions for the safe operation of natural gas pipeline in the conditions of its undermining during stope operations. The boundaries of the longwall face No. 1065 impact zone on the earth's surface and the unfermined gas pipeline section have been determined. The values of the earth's surface displacements and deformations in points along the gas pipeline route in the studied areas have been calculated.

Based on the determined conditions for safe undermining, it has been revealed that in the gas pipeline section between the PK212+40 and PK213+80 (140 m) pickets, the estimated parameters of the earth's surface horizontal deformations exceed their permissible values. Therefore, for the safe gas pipeline operation during the period of its undermining, in order to eliminate the hazardous impact of mining the longwall face No. 1065, additional protection measures must be applied.

It is recommended in the gas pipeline section between the PK212 and PK214+20 pickets that the gas pipeline be opened prior to the displacement process (at a distance of 200 m from the stoping face), thus reducing the density of the gas pipeline-soil system. The gas pipeline undermining, according to the given recommendations, should be controlled by instrumental observations. When opening a gas pipeline, based on the recommendations, it is expedient to conduct a visual inspection of its state during the displacement process active stage (preferably with an interval of 1–2 weeks).

## Data Availability

All data generated or analysed during this study are included in this published article.
